# Loss-of-function mutations in the melanocortin-2-receptor (*mc2r*) lead to skin hyperpigmentation in teleost fish

**DOI:** 10.1038/s41598-026-37998-7

**Published:** 2026-02-04

**Authors:** Elisa Barreiro-Docío, Laura Guerrero-Peña, Priyanka Soni, Luis Méndez-Martínez, Carolina Costas-Prado, Maria Victoria Alvarado, José Antonio Vázquez, Lluís Tort, José Miguel Cerdá-Reverter, Josep Rotllant

**Affiliations:** 1 Aquatic Biotechnology Lab, Instituto de Investigaciones Marinas, Consejo Superior de Investigaciones Científicas (IIM-CSIC), Vigo, Galicia Spain; 2 Department of Earth Science, University of Southern California, Los Angeles, CA USA; 3 Fish NeuroBehaviour Lab, Department of Fish Physiology and Biotechnology, Institute of Aquaculture from Torre la Sal (IATS-CSIC), Castellon, Spain; 4 Grupo de Reciclado y Valorización de Residuos (REVAL), Instituto de Investigacións Mariñas, Consejo Superior de Investigaciones Científicas (IIM- CSIC), Vigo, Galicia Spain; 5 Department of Cell Biology, Physiology and Immunology, Universitat Autonoma de Barcelona, Bellaterra, Spain

**Keywords:** Mc2r, Knockout, Melanocortin, Pigmentation, Steroidogenesis, Zebrafish, Cell biology, Developmental biology, Genetics, Molecular biology, Physiology

## Abstract

Melanocortins regulate pigmentation via melanocortin receptors (MCRs), which are highly conserved across vertebrates. Unlike other MCRs, the melanocortin 2 receptor (MC2R) is exclusively activated by ACTH; however, its role in pigmentation remains unclear. Using CRISPR/Cas9-generated *mc2r* knockout zebrafish, we demonstrated that the loss of *mc2r* in zebrafish results in impaired interrenal steroidogenesis and pronounced hyperpigmentation characterized by an increased number of melanophores and xanthophores while preserving normal patterning. Transcriptomic analyses revealed the upregulation of genes involved in melanosome formation, melanin synthesis, lipid metabolism, and carotenoid accumulation. These findings demonstrate that, in addition to controlling steroidogenesis, *mc2r* plays a key role in pigment cell development and metabolic regulation.

**Supplementary Information:**

The online version contains supplementary material available at 10.1038/s41598-026-37998-7.

## Introduction

Pigmentation in living organisms is essential for a number of vital survival functions, including photoprotection^1^, thermoregulation^2^ and camouflage^3,4^. The determination of the pigmentation pattern is influenced by a combination of genetic and environmental factors and is driven by specialized pigment cells derived from the neural crest^5^. In vertebrates, the formation of pigment patterns involves diverse pigment cell types. In mammals and birds, melanocytes are the only cells responsible for producing eumelanin or pheomelanin^6,7^. In contrast, other vertebrates, such as amphibians, reptiles and fish, possess a greater variety of chromatophores, including melanophores, xanthophores, erythophores and iridophores^8^.

Among vertebrates, zebrafish (*Danio rerio*) constitute a model organism for the study of pigmentation systems, particularly stripe formation. This striped pattern results from the interplay of two primary mechanisms. The first mechanism, highly conserved across evolution^9^, is regulated by the agouti signaling protein (Asip), which controls dorsoventral countershading. This pigmentation pattern results in a darker dorsal (upper) body surface and a lighter ventral (lower) surface^10^. The resulting gradient provides camouflage, helping animals blend with their environment by counteracting light and shadow effects. Asip modulates melanocyte melanin production, influencing the balance between eumelanin (dark pigment) and pheomelanin (light pigment)^10^.

The second mechanism, responsible for the alternating dark (blue) and light (yellowish) stripes, involves the interaction of three pigment cells: xanthophores, (orange pteridine pigments); melanophores (eumelanin), and iridophores (reflective guanine platelets)^11^. Melanophores congregate in dark stripes due to repulsion from xanthophores, while xanthophores populate the light stripes (interstripes), attracted by dense iridophores. Iridophores locally inhibit melanophore survival while promoting it at a distance^12,13^. They are present in both regions but differ in morphology: cubical in interstripes and stellate in stripes^14^. Iridophores are indispensable for stripe formation; in their absence, mutants exhibit only two rudimentary melanophore stripes that fragment into spots^15^. These interactions underscore the essential roles of the three pigment cell types in generating the characteristic zebrafish stripe pattern.

Melanocortins are peptides derived from proopiomelanocortin (POMC)^16^, including alpha-melanocyte-stimulating hormone (α-MSH) and adrenocorticotropic hormone (ACTH)^17^. They exert their effects by binding to melanocortin receptors (MCRs), a group of G protein-coupled receptors (GPCRs) distributed expressed across various tissues and involved in many physiological processes, including pigmentation regulation. Five melanocortin receptors have been identified and are highly conserved between humans and zebrafish^18^. Despite their structural and pharmacological similarities, these receptors differ in spatial distribution, physiological actions, ligand affinities and specificities in zebrafish.

Among the melanocortin receptors involved in pigmentation, melanocortin receptor 1 (Mc1r) is the most extensively characterized^19^. Mc1r is regulated by α-MSH and Asip1 and is expressed in the skin, where it plays a crucial role in regulating pigmentation. In mammals, Mc1r is primarily responsible for skin and hair pigmentation^20^. However, the functions of the remaining melanocortin receptors in pigmentation remain poorly understood.

Melanocortin receptor 2 (Mc2r) is exclusively activated by ACTH, and its functional expression requires the binding of the melanocortin receptor accessory protein (Mrap1)^21^. It is expressed predominantly in the adrenal gland, where it regulates glucocorticoid synthesis and secretion^18^. In fish, this includes cortisol production, which mediates stress response, inflammation suppression, food intake, and hormone regulation^22^. Although the role of Mc2r in the adrenal gland is well established, recent studies suggest that ACTH may also influence pigmentation by affecting chromatophores, possibly through melanosome dispersion, linked to background adaptation^23,24^. In mammals, Mc2r mutations cause hyperpigmentation in humans^25,26^ and pigmentation defects in knockout mice^27^,but its effects on pigmentatioin in fish remain unexplored.

Other melanocortin receptors, such as Mc3r and Mc4r, are expressed in the central nervous system and regulate energy balance. Mc5r may also play also a role in pigmentation in fish, as it is expressed in chromatophores alongside Mc1r^28,29^.

The objective of this study was to investigate the role of Mc2r in both steroidogenesis and in pigmentation. To this end, zebrafish mutants with a loss-of-function mutation in *mc2r* gene were generated via the CRISPR/Cas9 system. The *mc2r*^*KO*^ mutants were characterized through pigment cells counts, transcriptomic analysis of the skin, and assessment of steroidogenesis capacity via acute stress response experiments evaluating cortisol synthesis. Additionally, the proportions of different pigment cell types present in wild-type and mutant fish were estimated via bulk tissue deconvolution, using single-cell RNA-seq skin data as a reference. This approach enabled the examination of phenotypic alterations and transcriptional consequences in pigment-associated genes, provinding deeper insights into Mc2r’s role in pigmentation and its broader function in regulating steroidogenesis.

## Results

### Identification of nonfunctional mutations in the zebrafish *mc2r* gene

**Fig. 1:**
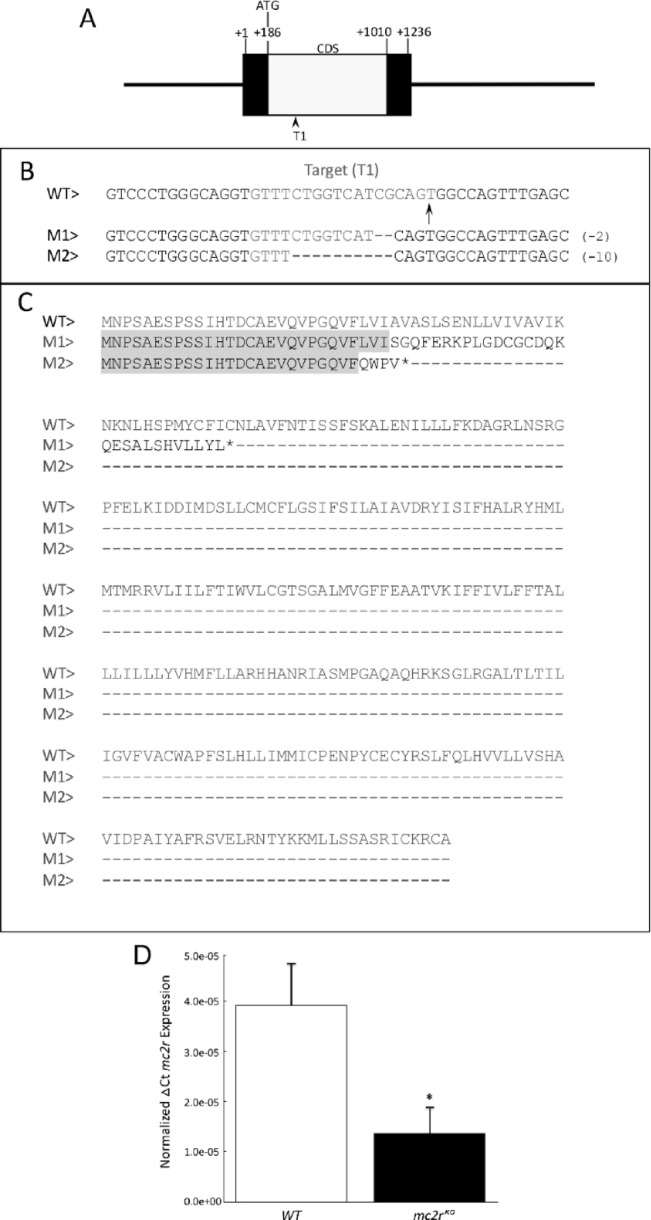
CRISPR/Cas9-induced mutations in the zebrafish *mc2r* gene. **(A)** Diagram of the *mc2r* single-exon gene. The coding region (CDS) is depicted as white boxes, whereas the 5′ UTR and 3′ UTR are shown as black boxes. The target site is represented by a black arrow. **(B)** Sequence of induced deletions in the *mc2r* locus. The first line represents the sequence of a WT zebrafish strain. The target sequence is highlighted in red. The black arrow indicates the protospacer-adjacent motif (PAM). The second and third lines show the different mutations that were induced. The second and third lines show the different mutations that were induced, scoring the deletion of each mutation on the right side. **(C)** Amino acid sequence of the complete protein of the zebrafish *mc2r* gene. The first line is the sequence of a WT fish, and the second and third lines represent the predicted sequences of both mutations. The gray background indicates the conserved sequence between the mutated and WT strains. Asterisks represent a codon stop. **(D)** Effects of CRISPR/Cas9-*mc2r* on *mc2r* mRNA. RT‒qPCR analysis of *mc2r* mRNA levels in wild-type (WT) (white bar) and mc2r knockout (*mc2r*^*KO*^) (black bar) fish. The results were normalized to *β-actin* (*actb*) and are expressed as the means ± SDs (*n* = 5 per group). *P < 0,05.

The zebrafish *mc2r* gene has a single exon, which results in the production of a single transcript of 1,263 base pairs in length that encodes a 304 amino acid (aa) protein (Fig. 1A). Loss-of-function mutations of *mc2r* were created by selecting one target site via the CRISPR/Cas9 system. The target sequence was selected 67 bp after ATG, resulting in different sequence mutations. Two of these mutations, designated M1 (CRISPR-*mc2r*.iim01) and M2 (CRISPR-*mc2r*.iim02), were identified as frameshift mutations through the loss of 2 bp (Del 269–271) and 10 bp (Del 261–271), respectively (Fig. 1B). Both mutant alleles of *mc2r*^*KO*^ result in the introduction of a premature stop codon. The *mc2r.iim01* allele encodes a truncated 57 aa protein, with the first aa matching those of the complete WT Mc2r protein. In contrast, *mc2r.iim02* encodes a protein of only 29 amino acids, 25 of which are shared with WT Mc2r (Fig. 1C). The two *mc2r*^*KO*^ lines presented a comparable increased pigmentation across the body and fins (image not shown in the manuscript). To ensure consistency in subsequent analyses, we focused primarily on the *mc2r.iim01* homozygous line (hereafter referred to as *mc2r*^*KO*^). We quantitatively assessed *mc2r*^*KO*^ efficiency at the mRNA level via RT‒qPCR (Fig. 1D). The observed CRISPR/Cas9-mediated effects on *mc2r* mRNA revealed a partial decrease in *mc2r* mRNA. Previously, frame-shift mutations resulting from nonhomologous end joining (NHEJ) DNA repair were shown to result in nonsense-mediated mRNA decay^30^.

### *mc2r* loss-of-function mutation impairs the steroidogenic response to acute stress

**Fig. 2:**
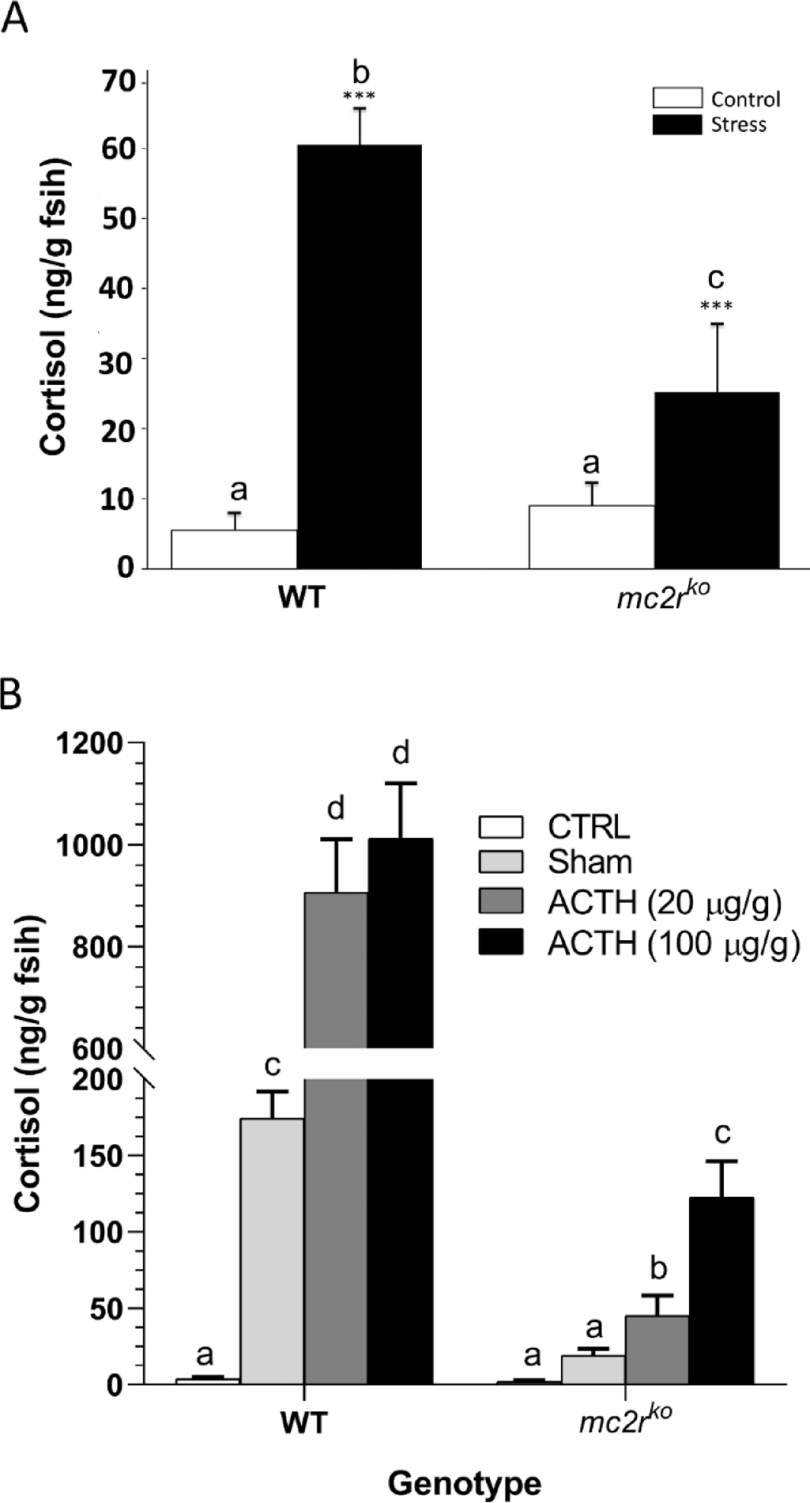
**(A)** Whole-body cortisol concentrations in wild-type (WT) and mc2r knockout (*mc2r*^*KO*^) zebrafish following acute stress exposure. Fish were subjected to repeated air exposure stress (3 × 2 min) and sampled 30 min after the final exposure. Cortisol was extracted and quantified using a radioimmunoassay (RIA) validated for zebrafish by Cortes et al., 2018. The data are presented as the means ± SEM (*n* = 20 per genotype per condition; 5 fish per tank, 4 tanks). Statistical analysis was performed via two-way ANOVA (*P < 0.05). Asterisks indicate significant differences between the control and stressed groups within the same genotype, whereas different letters denote significant differences between genotypes. **(B)** Effects of intraperitoneal injection of ACTH (20 and 100 µg/g body weight) in WT and *mc2r*^*KO*^ animals. Sham animlas were injected with saline whereas CTRL were not manipulated before euthanizing them in MS222 oversdose. Data represent the means ± SEM (*n* = 7 per treatment and genotype). Statistical analysis was performed via two-way ANOVA and different letters indicate significant differences at *p* < 0.05.

To see how alterations *mc2r* gene affect the response to stress, we measure the cortisol levels produced. There was no significant difference in basal cortisol levels between WT and *mc2r*^*KO*^ fish (Fig. 2A). Following acute stress, WT individuals exhibited a robust steroidogenic response, with whole-body cortisol levels increasing almost tenfold compared with those under control conditions (from approximately 6 ng/g to approximately 60 ng/g of fish; ***p < 0.001). In contrast, the *mc2r*^*KO*^ mutants presented a significantly blunted response, with cortisol levels increasing to only ~ 25 ng/g in the fish under stress conditions (***p < 0.001 versus the control), which was markedly lower than the stress-induced levels observed in the WT fish (***p < 0.001). To further corroborate the *mc2r*^*KO*^ mutants phenotype, we injected ACTH intraperitoneally into both genotypes (Fig. 2B). Saline injection alone promoted a significant increase in whole-body cortisol levels in WT animals only. Both ACTH doses (20 and 100 µg/g) promoted a dramatic increase in cortisol levels in WT fish, which was also evident in *mc2r*^*KO*^ mutants. However, ACTH-stimulated whole-body cortisol levels only reached approximately 5% (20 µg/g) and 12% (100 µg/g) of the levels exhibited by WT animals that were injected. These results demonstrate that Mc2r function is required for appropriate cortisol synthesis and secretion in response to acute stress, confirming its essential role in regulating steroidogenesis.

### *mc2r* loss-of-function mutation induces hyperpigmentation

**Fig. 3:**
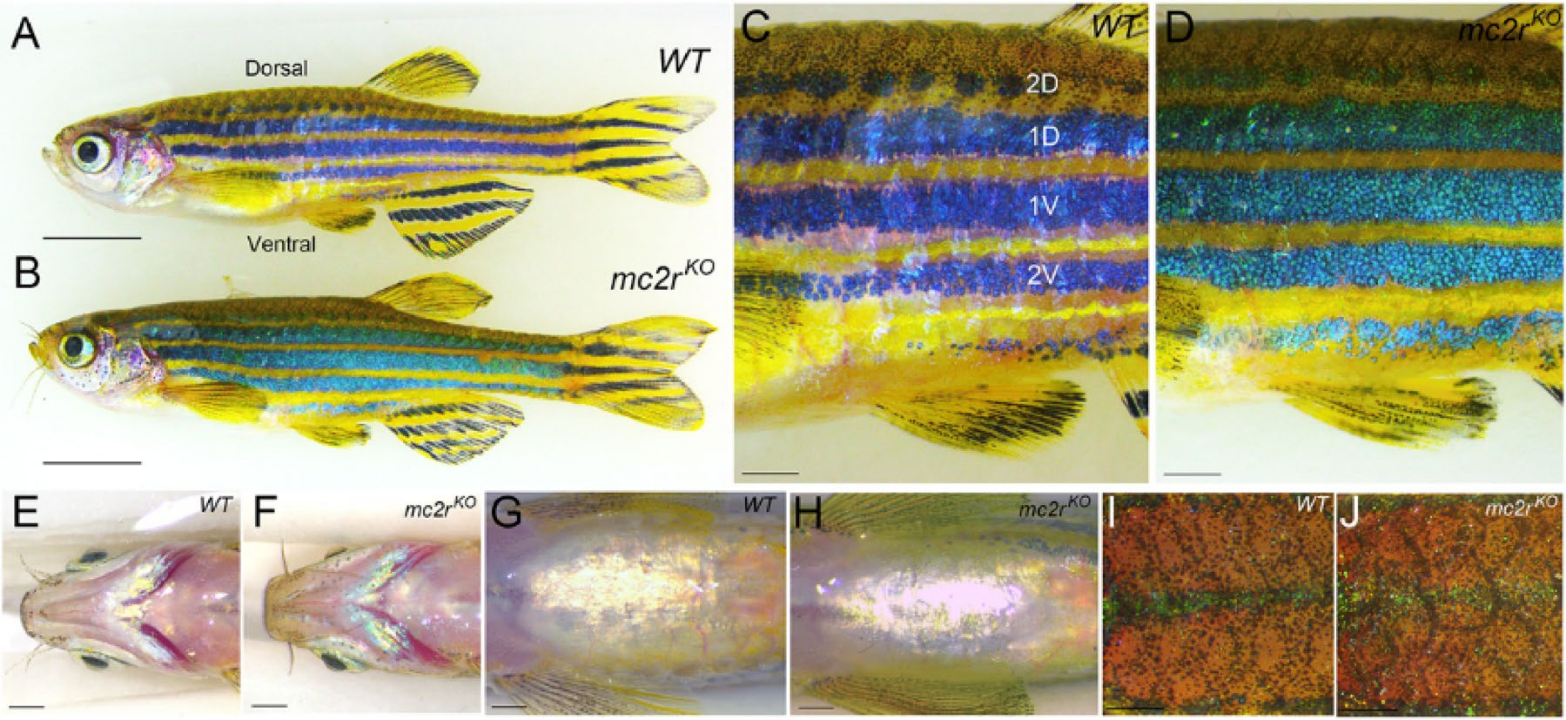
Hyperpigmented phenotype of *mc2r*^*KO*^ fish. Lateral **(A**,** B)**, posterior-lateral **(C**,** D)**, ventral head **(E**,** F)**, ventral belly **(G**,** H)** and dorsal **(I**,** J)** views of WT **(A**,** C**,** E**,** G**,** I)** and *mc2r*^*KO*^ mutant **(B**,** D**,** F**,** H**,** J)** fish. Zebrafish have a characteristic striped pigment pattern: 1D and 1V are the nomenclature used for primary stripes, and 2D and 2V are the nomenclature used for secondary stripes. Scale bars: **(A**,** B)** 5 mm, **(C-J)** 1 mm.

**Fig. 4:**
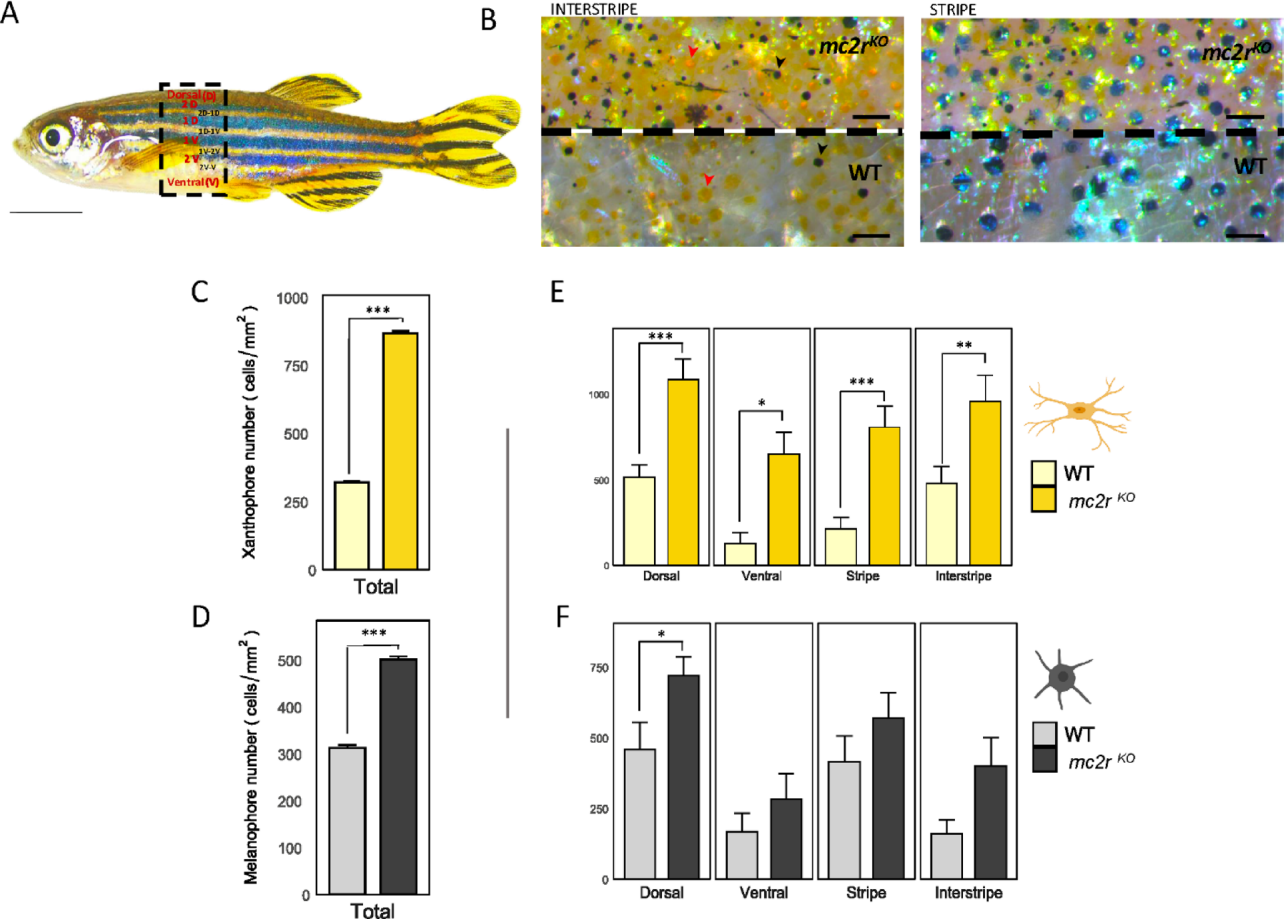
Dorso-ventral and stripe-interstripe distributions of melanophore and xanthophore cells in WT and *mc2r*^*KO*^ fish. **(A)** Lateral view of a zebrafish highlighting the selected stripes (2D, 1D, 1V, 2V), interstripe (2D-1D, 1D-1V, 1V–2V, 2V-V), dorsal (D) and ventral (V) regions in red, and the interstripe areas for cell counting in black. **(B)** Density and distribution of melanophores (black arrowhead) and xanthophores (red arrowhead) in an interstripe (left) of *mc2r*^*KO*^ mutant (top) and WT fish (bottom) and a stripe (right) of *mc2r*^*KO*^ mutant (top) and WT fish (bottom). **(C)** Total number of xanthophores/mm^2^ over the entire lateral flank of the wild type (light yellow) and the *mc2r*^*KO*^ mutant (dark yellow). **(D)** Total number of melanophores/mm^2^ over the entire lateral flank of the WT (gray) and the *mc2r*^*KO*^ mutant (black). **(E)** Quantification of xanthophores in the dorsal, ventral, stripe and interstrip regions in the WT (light yellow) and *mc2r*^*KO*^ mutant (dark yellow) strains. **(F)** Number of melanophores/mm^2^ in the dorsal, ventral, striped and interstripe regions in the WT (gray) and *mc2r*^*KO*^ mutants (black). The data are presented as the means ± SEMs, *n* = 3. Asterisks indicate significant differences between the WT and the *mc2r*^*KO*^ mutant. Two-tailed Student’s t test was used to compare different body stripes between WT and *mc2r*^*KO*^ fish to determine the significant differences in pigments (*, *P* < 0.05; **, *P* < 0.01; ***, *P* < 0.001). Scale bars: (A) 5 mm, (B) 2 mm. The cell figures were provided by BioRender.com.

Compared with their WT siblings, all the homozygous *mc2r*^*KO*^ lines displayed notable hyperpigmentation, characterized by a more generally yellowish appearance (Fig. 3A, B). The stripes of *mc2r*^*KO*^ mutant fish lost their characteristic WT intense black color, resulting in a bluish-greenish hue (Fig. 3C, D). Additionally, melanophores were observed in atypical locations where they are usually less abundant, such as around the jaws (Fig. 3E, F). Similarly, xanthophores were more abundant than usual in the belly region. Moreover, the interstripe regions of both the ventral (Fig. 3G, H) and dorsal (Fig. 3I, J) areas appeared more yellowish in *mc2r*^*KO*^ individuals than in their WT siblings. However, despite the discovery of pigment cells in less frequent locations, the distinctive striped pigmentation pattern of zebrafish remained intact in *mc2r*^*KO*^ individuals. To characterize this phenotype further, additional images and quantifications in Fig. 4 illustrate the distribution and density of pigment cells across different skin regions.

### Pigment distribution in hyperpigmented fish resulting from loss-of-function mutation of the *mc2r* gene

The number of xanthophore and melanophore cells was quantified in both WT and *mc2r*^*KO*^ (Fig. 4A), distinguishing between stripes and interstripes, as well as between the dorsal and ventral areas (Fig. 4B). This analysis aimed to determine the abundance and distribution of pigment cells in *mc2r*^*KO*^ mutant fish. Cell counting confirmed a general increase in the number of melanophores and xanthophores in the *mc2r*^*KO*^ mutant (Fig. 4C,D). Specifically, the number of xanthophores was significantly greater in *mc2r*^*KO*^ mutants (898.523 xanthophores/mm²) than in WT fish (360.633 xanthophores/mm², *P =* 0.0002; Fig. 4C). Similarly, the number of melanophores was greater in *mc2r*^*KO*^ mutant fish (501.133 melanophores/mm²) than in WT fish (312.578 melanophores/mm², *P =* 0.009; Fig. 4D). With respect to the dorsal-ventral distribution of pigment cells, a considerable increase in xanthophores was observed in both the dorsal (*P =* 0.003) and ventral (*P =* 0.023) regions of *mc2r*^*KO*^ fish compared with WT fish (Fig. 4E). In contrast, a significant increase in melanophores was observed only in the dorsal region (*P =* 0.0323) in *mc2r*^*KO*^ fish (Fig. 4F), whereas the characteristic dorsoventral countershading pattern remained intact. In zebrafish, melanophore-rich dark stripes alternate with xanthophore-dominated light interstripes, as evidenced in WT fish (Fig. 4E,F). In the *mc2r*^*KO*^ mutants, despite a significant increase in the number of pigment cells on both stripes (*P =* 0.004) and interstripes (*P =* 0.012) (Fig. 4E,F), the overall stipe pattern remained intact. Notably, in the ventral region of *mc2r*^*KO*^ fish, melanophores accumulated more prominently around the mandibular area, whereas xanthophores were more abundant in the belly. This increased presence of xanthophores did not disrupt the striped pattern but rather altered its spatial distribution, resulting in a broader dispersion of yellow pigmentation across the skin.

### The expression profile of pigmentation-specific genes is affected by the *mc2r* gene mutation

Loss of function of *mc2r* has been shown to result in hyperpigmentation in zebrafish, according to a cell counting-based analysis (see above). Consequently, to determine the differences in the expression profiles of pivotal genes associated with pigmentation between the *mc2r*^*KO*^ and WT fish, a skin-specific transcriptome analysis was conducted on both the *mc2r*^*KO*^ and WT fish. More than 20 million paired-end reads of 150 base pairs were generated for each sample. Following quality filtering, the clean reads were mapped to the zebrafish reference genome, with average mapping rates of 93.24% and 93.42% for the WT and *mc2r*^*KO*^, respectively. Two-by-two comparisons were performed between WT and *mc2r*^*KO*^ to identify possible variations in the expression profiles of pigmentation-related genes. A total of 2040 differentially expressed genes (DEGs) were identified, with 955 and 1085 genes exhibiting increased expression in *mc2r*^*KO*^ and WT, respectively (Supplementary Table 1). A functional gene ontology (GO) analysis of the aforementioned genes revealed a statistically significant enrichment of molecular functions, including protein tyrosine/threonine phosphatase activity (GO:0008330), MAP kinase tyrosine phosphatase activity (GO:0033550), and MAP kinase tyrosine/serine/threonine phosphatase activity (GO:0017017) (see Supplementary Table 2). Conversely, Kyoto Encyclopedia of Genes and Genomes (KEGG) pathway analysis revealed enrichment of the tyrosine metabolism pathway (dre00350), which identified pivotal genes associated with differential pigmentation expression, including *tyr*,* aox5*,* tyrp1a* and *tyrp1b*. Furthermore, an enriched pathway related to cytoskeletal dynamics in muscle cells (dre04820) was also observed.

### Loss-of-function mutations in *mc2r alter* the expression of specific genes involved in pigmentation

**Fig. 5:**
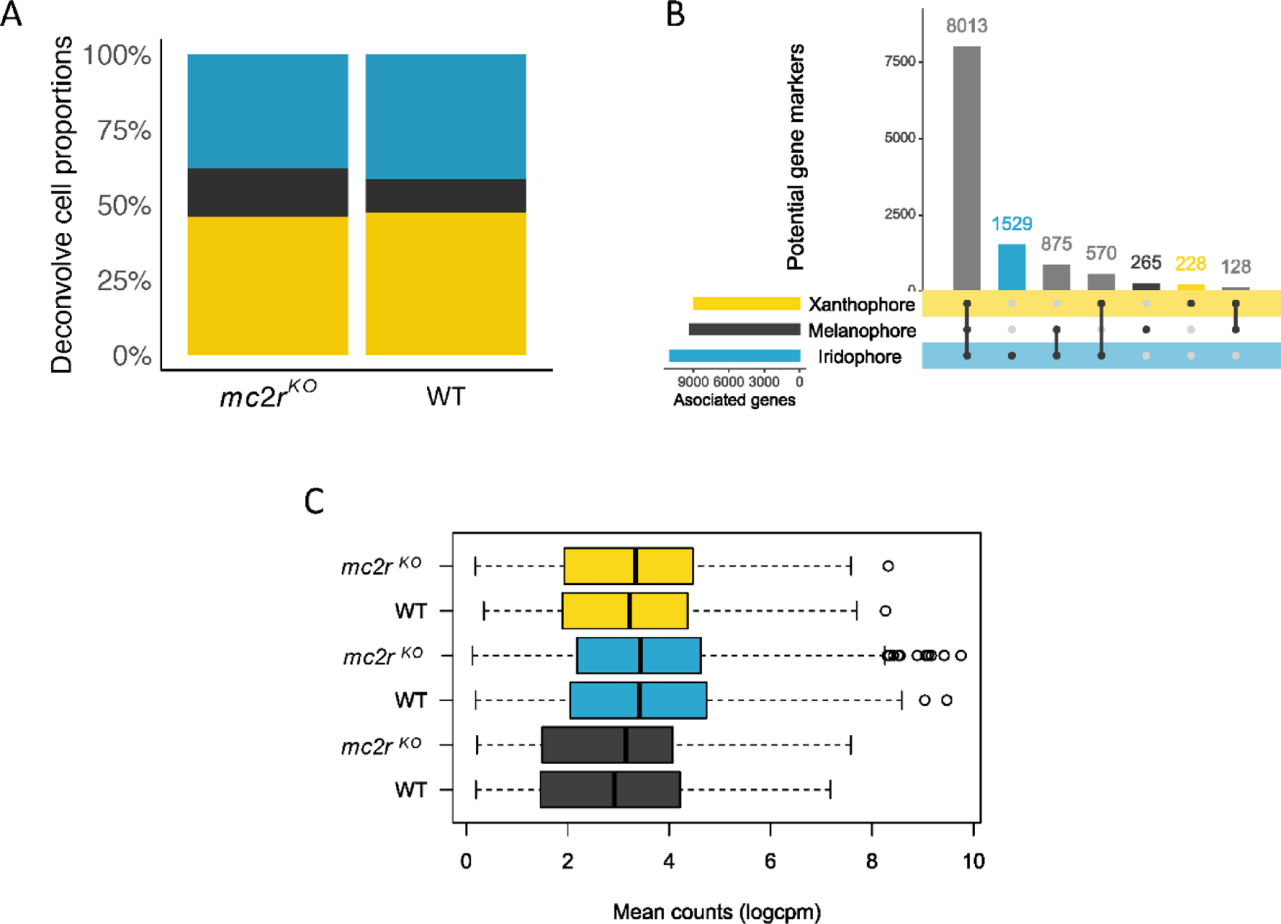
Pigment cell distribution and differential gene expression in *mc2r*^*KO*^ mutant and WT zebrafish skin. **(A)** Estimation of the proportion of pigment cells in the skin of *mc2r*^*KO*^ and WT fish via deconvolution analysis of bulk transcriptome data, with single-cell data used as a reference. **(B)** UpSet plot demonstrating significantly background-dependent differentially expressed genes (DEGs) from skin single-cell transcriptome data^63^ for xanthophore, melanophore, and iridophore cells. Distinctively expressed genes for iridophores, melanophores, and xanthophores are indicated with blue, black, and yellow bars, respectively. **(C)** Box plot of the counts of pigment cell types in WT and *mc2r*^*KO*^ skin, based on DEGs unique to each pigment cell type, as identified in the UpSet plot. The black lines represent the median, the whiskers indicate the 25th and 75th percentiles, and the points represent outliers. Melanophores are represented in black, xanthophores in yellow, and iridophores in blue.

A WT zebtafish single-cell skin-specific transcriptome reference (GSM7029635) was employed to deconvolve pigment-cell type proportions in *mc2r*^*KO*^ and WT from skin bulk transcriptomic data. Our analysis demonstrated that *mc2r* does not have a specific target pigment, as the relative abundances observed among melanophores, xanthophores, and iridophores were comparable between WT and *mc2r*^*KO*^ (Fig. 5A). The estimated proportions of pigment cell types in *mc2r*^*KO*^ were 16.06%, 46% and 38.04% for melanophores, xanthophores and iridophores, respectively. Moreover, for the WT fish, the percentages of melanophores, xanthophores and iridophores were 11.15%, 47.32% and 41.52%, respectively. On the basis of these data, we identified 265 potential marker genes for melanophores, 228 for xanthophores and 1,529 for iridophores (Fig. 5B). However, despite these phenotypic differences, no significant differences in the total gene counts of the potential markers were detected between the *mc2r*^*KO*^ and WT fish (Fig. 5C).

**Fig. 6:**
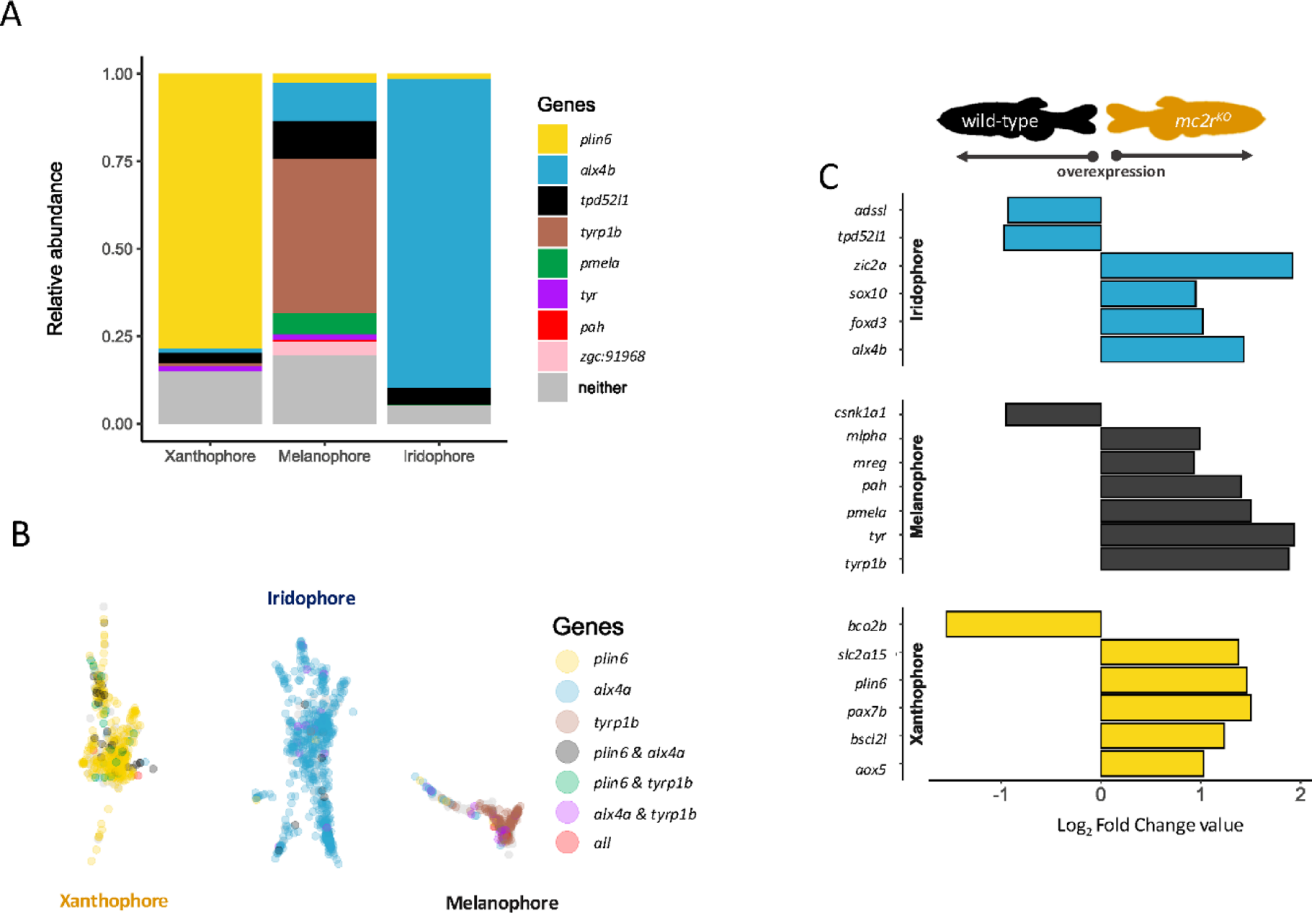
Gene expression analysis and visualization of potential marker genes in WT pigment cell clusters. **(A)** Proportion of cells in WT skin expressing a specific set of genes within each pigment cell cluster. “Neither” denotes the proportion of cells in the cluster that do not meet the minimum threshold for the expression of selected genes. **(B)** Uniform manifold approximation and projection (UMAP) visualization of pigment cell clusters from WT fish. The data are represented by dots, each corresponding to a single cell, with cells expressing specific genes or coexpressing several genes color coded. **(C)** Fold change values of differential expression between the WT and the *mc2r*^*KO*^ mutant for marker genes identified in each pigment cell type. A negative fold change value indicates overexpression in the WT, whereas positive values denote overexpression in the *mc2r*^*KO*^ mutant. All the genes displayed a p value of less than 0.05.

To validate the cell type specificity of our differentially expressed genes from the bulk transcriptome, we employed single-nuclei transcriptomes of WT skin and analyzed melanophore (324 cells), xanthophore (535 cells) and iridophore (776 cells) populations. Specific pigment genes were identified, including *plin6* for xanthophores, *alx4b* for iridophores and *tyrp1b* for melanophores (Fig. 6A) (marker gene expression data are available in Supplementary Table 3). Specifically, we found that *plin6* is predominantly expressed in xanthophores, whereas *alx4b* and *tpd52l1* appear to be more highly expressed in iridophores. In melanophores, *tyrp1b* and *pmela* are expressed in the majority of cells, along with other genes, such as *pah*, *tyr*, and *mreg* (Fig. 6B). Notably, the genes identified as potential cell type-specific markers exhibited differential expression between the control and mutant fish (Fig. 6C). Therefore, we identified a number of genes that are differentially expressed between *mc2r*^*KO*^ and WT and are associated with melanophore cells, including *pmela*, *pah*, *tyr* and *tyrp1b*. Similarly, we observed differential expression of the *bscl2l*, *plin6*, *slc2a15* and *bco2b* genes in xanthophores, whereas *alx4b* and *zic2a* were highly expressed in iridophores.

## Discussion

The pigmentation of zebrafish is dependent on the interaction of multiple genes and environmental factors. In fish, the melanocortin activity is mediated by five different receptors, and it is well documented that at least the *mc1r* gene is involved in coloration^10^. The *mc2r* gene, which is expressed in the adrenal gland and is involved in adrenal biosynthesis, has also been linked to pigmentation. Studies in mice and humans have shown that alterations in this receptor can lead to pigmentation disorders^25–27^. These findings highlight the potential role of mc2r in pigmentation regulation, although the involvement and potential regulatory mechanisms of mc2r in fish pigment patterns remain unclear^31–33^. This study aimed to elucidate the impact of mc2r on pigmentation in zebrafish through knockout experiments (*mc2r*^*KO*^), with a focus on pigment cell distribution, abundance and gene expression changes. By integrating cell counting data with published skin single-cell transcriptomic data and whole-skin tissue bulk transcriptomes from both WT and *mc2r*^*KO*^ fish. In addition to pigmentation, our study highlights the critical role of mc2r in the steroidogenic response to acute stress. While basal cortisol levels were comparable between WT and *mc2r*^*KO*^ fish, the stress-induced increase in cortisol was severely blunted in the mutants, indicating a marked impairment in their capacity to synthesize cortisol upon acute challenge. Moreover, ACTH-challenge experiments further validated this deficiency: although ACTH administration triggered a robust rise in whole-body cortisol in WT fish, *mc2r*^*KO*^ mutants exhibited only minimal increases, reaching approximately 5% and 12% of WT cortisol levels following 20 µg/g and 100 µg/g ACTH injections, respectively. This phenotype is consistent with the established role of mc2r as the ACTH receptor, which is essential for initiating adrenal steroidogenesis^21^. This sharply reduced responsiveness confirms that Mc2r is strictly required for efficient cortisol synthesis and secretion during acute stimulation. Together, these findings corroborate the functional loss of mc2r in steroid physiology and suggest that pigmentation alterations in the mutants occur in parallel with systemic endocrine dysregulation. These results provide evidence that mc2r simultaneously regulates pigmentation and steroidogenesis, underscoring its dual role in fish physiology.

In general, hyperpigmentation and a more yellowish appearance were observed in the *mc2r*^*KO*^ mutant fish than in the WT fish. Despite these alterations, the characteristic striped pattern of zebrafish (formed by the interaction of xanthophores, melanophores and iridophores^34–36^ remained intact. This finding suggests that while mc2r influences pigment cell numbers, it does not disrupt the underlying spatial area; a notable increase in melanophores was observed in the dorsal region, and the dorsoventral gradient, which typically results in a darker dorsal side and a lighter ventral side, is preserved. This finding is corroborated by the unchanged expression of *asip1*, a key regulator of dorsoventral countershading^37^, which is known to be an antagonist of Mc1r and Mc4r^38,39^. Therefore, despite the alteration of *mc2r*, dorsoventral countershading remains unaffected.

To investigate why the stripe pattern was not disrupted, we examined iridophore-related genes, since previous studies have demonstrated their pivotal role in pattern formation^14^. Key regulatory genes such as *tjp1a*, which is involved in interstripe iridophore formation and stripe maintenance^40^, were not differentially expressed between *mc2r*^*KO*^ and WT fish. Similarly, *tbx6*^41^, a gene whose alterations result in defects in interstripe development, was also not differentially expressed. These findings suggest that iridophore spatial organization remains unchanged in *mc2r*^*KO*^ mutant fish. Additionally, transcriptomic analysis revealed a balance in iridophore proliferation signals, with the upregulation of genes such as *alx4b*, which plays a role in iridophore development and differentiation^42^, and *zic2a*, which is associated with pigment cell proliferation and maintenance^43^, while *tpd52l1*, a proliferation inhibitor, was downregulated. This differential expression suggests a regulatory equilibrium wherein specific genes facilitate proliferation and others impede excessive growth, thereby ensuring a balanced distribution of pigment cells. This controlled proliferation may have contributed to the maintenance of the stripe pattern in the *mc2r*^*KO*^ mutant fish.

A comparative analysis of gene expression between *mc2r*^*KO*^ and WT samples, coupled with an investigation of identified marker genes, revealed that the observed increase in melanophores in *mc2r*^*KO*^ fish is associated with the overexpression of genes involved in melanosome and melanin synthesis (*pmela*,* mreg*,* tyr*,* tyrp1b*) and pigment granule distribution (*pah*). This may contribute to the hyperpigmented phenotype, as previously documented^44,45^. Furthermore, the overexpression of *mlpha*, which is implicated in pigment granule dispersion^46^, may contribute to a more uniform and extensive distribution of melanosomes, thereby further intensifying the darker phenotype. These findings indicate that Mc2r enhances the overall capacity for melanosome biogenesis and melanin production while maintaining spatial control over melanophore proliferation.

Conversely, a substantial increase in the number of xanthophore cells in the interstripes and stripes of both the dorsal and ventral regions was observed in *mc2r*^*KO*^ mutant fish. Unlike in WT fish, where xanthophores predominantly accumulate in interstripes, no statistically significant differences in xanthophore numbers between stripes and interstripes were found in *mc2r*^*KO*^ fish. These findings suggest that Mc2r plays a key role in regulating not only the total number of pigment cells but also their spatial distribution without disrupting the organization of the underlying spatial stripe pattern. The increase in xanthophore numbers across these regions, contributes to the more pronounced yellow phenotype observed in the mutants. Transcriptomic analysis revealed that the yellowish phenotype is linked to the overexpression of genes associated with lipid metabolism and carotenoid accumulation in xanthophores, such as *bscl2l* and *plin6*^47^. These genes likely promote the accumulation of lipid-soluble pigments, intensifying the yellow hue. Furthermore, the elevated expression of *slc2a15* indicates increased xanthophore metabolism, which may facilitate pigment accumulation^48^. The upregulation of *pax7b* and *aox5*, both of which are linked to xanthophore proliferation^49,50^, further supports this observation. Furthermore, the downregulation of *bco2b*, a gene involved in the conversion of carotenoids to apocarotenoids, may further increase carotenoid accumulation, intensifying the yellow phenotype^50^. Although the transcriptomic data strongly suggest a role for *mc2r* in xanthophore lipid metabolism, additional metabolic assays would be necessary to confirm these findings. Despite increased melanophore numbers in the dorsal region, no significant increase was detected in the ventral region apart from local accumulation in the mandibular area. The unchanged expression of genes involved in stripe formation further suggests that *mc2r* primarily regulates pigment cell abundance rather than altering their spatial organization. By combining a loss-of-function *mc2r* zebrafish mutant with a transcriptomic approach and deconvolution analysis based on a single-cell dataset, we were able to characterize the hyperpigmented phenotype observed in *mc2r*^*KO*^ mutant fish. This phenotype was driven primarily by an increase in melanophore and xanthophore numbers and a potential increased carotenoid accumulation in xanthophore cells. A key molecular signature of this phenotype is the enrichment of protein tyrosine/serine/threonine phosphatase and MAP kinase-related signaling pathways, which regulate pigment cell proliferation, differentiation, and survival. In particular, MAPK signaling plays a key role in melanogenesis and stress responses, suggesting its involvement in the pigmentation changes observed in *mc2r*^23,24^. Additionally, protein phosphatases help maintain signaling homeostasis, potentially balancing pigment cell expansion and metabolism^51^.

In support of the role of Mc2r in pigmentation, KEGG analysis revealed enrichment of the tyrosine metabolism pathway (dre00350), which includes key melanin biosynthesis genes. The upregulation of these genes is correlated with increased melanophore numbers in *mc2r*^*KO*^ mutants, reinforcing the role of Mc2r in pigment cell maintenance^8^. Interestingly, we also detected enrichment in a cytoskeletal dynamic pathway (dre04820). While primarily linked to muscle function, cytoskeletal remodeling could be crucial for pigment cell morphology, migration, and positioning. This could explain why, despite an increase in pigment cell number, the overall stripe pattern remains unchanged in *mc2r*^*KO*^ fish^41^. These findings highlight the hypothesis that Mc2r is a key regulator of pigmentation in zebrafish, influencing the development, distribution, and metabolic activity of pigment cells.

In conclusion, this study demonstrated that Mc2r plays a crucial dual role in zebrafish physiology, being essential not only for interrenal steroidogenesis but also for pigmentation control through the regulation of pigment cell development and distribution. Loss of *mc2r* function leads to impaired steroid hormone production and characteristic hyperpigmentation with a yellowish hue, driven by an increased number of melanophores and xanthophores. Despite these changes, the characteristic striped pattern and dorsoventral countershading remain intact, indicating that Mc2r influences pigment cell proliferation without disrupting overall pattern formation. The hyperpigmented phenotype is further supported by the upregulation of genes involved in melanosome biogenesis, melanin synthesis, and pigment granule transport, while the accumulation of carotenoids contributes to increased yellow coloration. Altogether, these findings highlight Mc2r as a key regulator of both steroidogenesis and pigmentation homeostasis, integrating endocrine and pigmentary pathways through its effects on cell number, gene expression, and pigment metabolism.

## Materials & methods

### Animals

The study was conducted using wild-type (WT) zebrafish of the TÜ strain (Tübingen, *Danio rerio*, Nüsslein-Volhard Laboratory) cultured and raised at the facilities of the Institute of Marine Research in Vigo (IIM-CSIC). All methods are reported in accordance with the ARRIVE guidelines (https://arriveguidelines.org). The Institutional Animal Care and Use Committee of IIM-CSIC approved this study in accordance with the Royal Decree (53/2013) and European Directive (2010/63/EU), as well as guidelines from the National Advisory Committee for Laboratory Animal Research (RD53/2013), under license from the Spanish Authority for the Protection of Experimental Animals (Reference: ES360570202001/18/FUN. 01/BIOL AN. 08/JRM).

### Generation of *mc2r* knockout mutants (*mc2r*^*KO*^)

The *mc2r* knockout mutant (*mc2r*^*KO*^) was generated via the CRISPR-Cas9 gene editing system following a previously described protocol^52^. The target sequence was identified with the ChopChop web tool^53^. Two oligonucleotides were used for PCR to obtain specific DNA fragments that included a target site sequence (5´-GGTGTTTCTGGTCATCGCAG-3´): a scaffold oligo xxxx The PCR was performed using Phusion High Fidelity PCR Master Mix Buffer (New England Biolabs, UK). The PCR products were purified via a DNA Clean & Concentration-5 Kit (Zymo Research, USA) according to the manufacturer’s instructions. The purified PCR product served as a template for in vitro transcription with the MEGAscript T7 High-Yield Transcription Kit (Ambion, USA) following the manufacturer’s instructions. The resulting gRNA was purified with RNA Clean&Concentrator 5 (Zymo Research, USA) and injected at a concentration of 25 ng/µL along with Cas9 mRNA (transcribed from the linearized pT3TS-nCas9n plasmid) at 50 ng/µL and phenol red solution (0.2). Approximately 2 nL of this mixture was microinjected into the cytoplasm of zebrafish eggs at the one-cell stage. The microinjection was performed via a dissection microscope (MZ8, Leica) equipped with an MPPI-2 pressure injector (ASI systems). Different mutations were induced and genotyped via PCR via the following primers: TS-F, 5’-TGCATCGATCTGCACATTGC-3’; reverse primer (TS-R), 5’-TGGCGAGGATGCTGAAGATT-3’. Two potential nonfunctional alleles were identified, and homozygous lines of each mutation were obtained by inbreeding.

### RT‒qPCR analysis

Five male fish (240 dpf) of the WT strain and *mc2r*^*KO*^ were sacrificed with 0.004% tricaine methanesulfonate MS-222 (Sigma Aldrich, Saint Louis, USA). The skin tissues of the top two stripes of the dorsal dermis region and the bottom two ventral stripes were dissected for *mc2r* expression analysis. RNA extraction was performed with an RNeasy Mini Kit (Qiagen, USA) following the manufacturer’s instructions, and the samples were treated with DNAse digestion solution (Qiagen, USA). cDNA was reverse transcribed with 100 ng of total RNA via the Maxima First Strand cDNA Synthesis Kit (Thermo Fisher Scientific, USA). The samples were amplified in duplicate, containing 10 µL of PowerUp SYBR Green Master Mix (2x) (Thermo Fisher Scientific, USA), 1 µL of 0.5 µM each primer, 6 µL of nuclease-free water, and 2 µL of cDNA template. The qRT‒PCR products were analyzed with a QuantStudio3 Real-Time PCR System (Thermo Fisher Scientific, USA). Gene expression of *mc2r*was assessed in two independent experiments via the efficiency-calibrated method of ΔCT described in Pfaffl W^54^.Relative mRNA expression levels were normalized to those of the housekeeping gene β-actin (*ACTB*), which was used as an endogenous reference. The primer sets used for *mc2r* expression were 5’CAATCTTCAGCATCCTCGCC3’ and 3’CCATTAGGGCTCCACTGGTG5’.

### Mutant phenotypic evaluation on the basis of cortisol levels

Four tanks of wild-type (WT) [body weight (BW) = 0.30 ± 0.011 g] and *mc2r* knockout (*mc2r*^*KO*^) fish (BW = 0.37 ± 0.013) (*n* = 5 per tank) were designated as either control or stress-exposed for each genotype. Following the protocol described by Ramsay et al.^55^, all the fish in the stressed tanks (WT and *mc2r*^*KO*^) were subjected to repeated air exposure, three iterations of 2 min each, before being returned to their respective tanks. Thirty minutes after the final exposure, fish were euthanized using tricaine methanesulfonate (MS-222; Sigma Aldrich, St. Louis, USA) at a concentration of 0.004% and immediately stored at − 80 °C. Control fish were euthanized without air exposure and stored at − 80 °C until cortisol extraction and concentration analysis.

For ACTH inyection experiments, a total of 8 tanks of WT and *mc2r*^*KO*^ fish (*n* = 7) were classified as control (CTRL), sham, ACTH20 and ACTH100. Animals were acclimated for 1 week to the new rearing conditions and then injected at 10 am with 1.4 µl of saline 0.85% (sham), 1.4 µl of ACTH 5 µg/µl to reach 20 µg/g (ACTH20) and 1.4 µl of ACTH 25 µg/µl to reach 100 µg/g (ACTH100). After 1 h, animals were rapidly euthanized as before and whole bodies stored at −80 until analyzed.

Cortisol were extracted and quatified via radioimmunoassay (RIA) as described by Cortes et al. 2018^56^, and validated for use in zebrafish.

The data were analyzed via GraphPad Prism version 8.0.1. Statistical comparisons were performed via two-way ANOVA, with significance set at *P* < 0.05. Statistically significant differences are indicated in the figures via symbols and letters (*, **, ***, *** and a,b,c,d). Asterisks denote significant differences between groups at specific time points (*P < 0.05; **P < 0.01; ***P < 0.001), whereas different letters (a,b,c,d) indicate significant differences among groups (*P* < 0.05). The results are presented as the means ± standard error of the mean (SEM).

### Mutant phenotypic evaluation on the basis of cell pigment counting

The pigmentation pattern of mc*2r*^*KO*^ mutant fish was examined and compared with that of WT fish (*n* = 3 for each genotype) by quantifying the number of xanthophores and melanophores. A total of ten different skin regions (1 mm^2^) were selected from 240 dpf (days post-fertilization) fish: dorsal (D), second dorsal (2D), interstripe 2D-1D, first dorsal (1D), interstripe (1D-1V), first ventral (1V), interstripe (1V–2V), second ventral (2V) and interstripe (2V-V), ventral (V). The fish were anesthetized with MS-222 (0.003%) (Sigma‒Aldrich, Saint Louis, USA) prior to immersion in epinephrine (10 mg/ml) (Sigma‒Aldrich) for a period of 15 min to facilitate melanosome contraction. The skin regions were photographed via a Leica M165FC stereomicroscope equipped with a Leica DFC310FX camera (Wetzlar, Germany). The microscope included a reference scale that allowed us to determine the actual dimensions of the observed area. Since the images contained a built-in measurement scale, we used this reference to crop each image to a standardized 1 mm² area for analysis. The quantification of melanophore and xanthophore cells was performed automatically via ImageJ software. The data were then statistically compared via Student’s t test (*P <* 0.05).

### Tissue collection and RNA isolation for sequencing

Three independent replicates per genotype were selected and euthanized with a lethal dose of MS-222 (0.1%) (Sigma‒Aldrich, USA). A total of six adult zebrafish were dissected by peeling the skin from the head to the caudal tail. A maximum of 30 mg of skin tissue was used for total RNA extraction from each individual via the RNeasy Mini Kit (QIAGEN GmbH, Germany) in accordance with the manufacturer’s instructions. The integrity of the extracted RNA was verified to ensure that the RNA integrity number (RIN) was greater than 8. Strand-specific mRNA libraries were then sequenced on an Illumina NovaSeq X Plus platform at Novogene Co. (China), generating paired-end reads of 150 base pairs (bp) in length (23 M reads on average).

### Quality control, read mapping and differential expression

Quality control of the raw RNA-seq data was performed via FastQC v0.12.0^57^. High-quality clean data were obtained by removing adapters and low-quality reads via fastp v0.20.0^58^. Only reads longer than 100 bp were aligned to the *Danio rerio* reference genome (GCA_000002035.4) via STAR v2.7.9a^59^, followed by gene count assignment via HTseq v0.10.0^60^. Genes with low counts were excluded from further analysis via the *filterByExpr* function in the R package edgeR v4.0.16^61^. Normalization of the count matrix was achieved via the trimmed mean of M values method. A linear general regression model was fitted to the data distribution with the edgeR function *glmFit*. Differential gene expression between *mc2r*^*KO*^ and WT was assessed through pairwise comparisons via the *exactTest* function from edgeR, and p values were adjusted with the *decideTest* function according to the Benjamini‒Hochberg correction method. Genes were determined to be differentially expressed (DEGs) if they presented a p value lower than 0.05 and a log_2_-fold change value greater than 1 in absolute value. Gene Ontology (GO) enrichment analysis was performed via the ClusterProfiler R package v4.10.1^64^. The genome-wide annotation for zebrafish was sourced from the org.Dr.eg.db v3.18.0 package. Similarly, KEGG analysis was performed utilizing the same packages to identify enriched terms and pathways.

### Relative abundance of pigment cell types

To ensure the reliability of our single-cell reference data, we used high-quality WT single-cell skin data^51^ to perform deconvolution analysis. The data were preprocessed to remove low-quality cells and doublets. Cell type annotations were validated through established marker gene expression data. Prefiltered data identified a total of 35,114 cells, annotating 43 different clusters that were assigned to 33 unique cell types. The raw count matrix of all identified cells (GSM7029635)^63^ was filtered to include only the specific transcriptomes of pigment cells: melanophore (*n* = 324), xanthophore (*n* = 535) and iridophore (*n* = 776) cells. We subsequently conducted a deconvolution analysis via the Multi-Subject Single-cell tool (MuSiC v.1.0.0)^64^. This tool is optimized for tissues with closely related cell types, employing a probabilistic model along with weighted nonnegative least squares regression to estimate the relative abundance of pigment cell types from bulk transcriptomic data^64^. We also validated our findings by comparing the deconvolution results with the gene expression patterns of known marker genes. Default settings were used for the deconvolution analysis. Using the processed single-cell RNA-seq data as a reference and our bulk skin transcriptomic data, we estimated pigment cell-type proportions in *mc2r*^*KO*^ and WT zebrafish.

### Identification of the cell type specificity of DEGs between *mc2r*^*KO*^ mutant and WT fish

The differentially expressed sets of genes for each pigment cell population in the WT were selected from the data matrix of the transcriptomic single-cell study conducted by Aman et al^65^. (GSM7029635). We identified groups of genes that were uniquely differentially expressed for each pigment cell type, with the aim of obtaining potential marker genes for melanophores, xanthophores and iridophores. This was achieved via UpSetR v1.4.0 software^65^. The transformed counts for the genes previously identified as specific to each cell type were explored to gain insight into the bulk skin transcriptomic average. The transformed counts were subsequently visualized in a boxplot. The statistical significance between the different fish conditions and pigment cells was determined via a t test. Furthermore, a UMAP plot (Uniform Manifold Approximation and Projection for Dimension Reduction) was employed to illustrate the differential expression of genes between *mc2r*^*KO*^ mutants and WT. This analysis was conducted with WT single-cell data as a reference point to ascertain cell-type specificity, employing the monocle3 R package (version 1.3.7)^66^. To provide additional insight, a bar plot was constructed to represent the same results as the UMAP visualization, illustrating the proportion of marker genes present in each cell cluster. Furthermore, a log_2_-fold change (logFC) bar plot was generated to display several marker genes according to their cell specificity.

## Supplementary Information

Below is the link to the electronic supplementary material.

Supplementary Material 1

Supplementary Material 2

Supplementary Material 3

Supplementary Material 4

## Data Availability

The datasets generated and/or analysed during the current study are available in the NCBI’s Gene Expression Omnibus (GEO) repository, [https://www.ncbi.nlm.nih.gov/geo/query/acc.cgi and Accession Number: GSE292242] and are also available from the corresponding author upon reasonable request.
