# Supplementary material for: Loss-of-function mutations in the melanocortin-2-receptor (mc2r) lead to skin hyperpigmentation in teleost fish
Source: Sci Rep. 2026 Feb 4;16:7261. doi: 10.1038/s41598-026-37998-7 (PMC12923660; doi:10.1038/s41598-026-37998-7)
Supplement: Supplementary file 4 — Supplementary Material 4 [file 41598_2026_37998_MOESM4_ESM.docx]

**Supplementary Tables**

**Supplementary Table 1.** List of differentially expressed genes between *mc2r* knockout (*mc2r^KO^*) and wild type (WT) zebrafish. The table includes Ensembl gene IDs, log fold change (logFC), log counts per million (logCPM), *P*-value, and false discovery rate (FDR). Positive logFC values indicate upregulation in *mc2r^KO^* relative to WT, whereas negative values indicate downregulation.

**Supplementary Table 2.** Results of Gene Ontology (GO) enrichment analysis of differentially expressed genes between *mc2r* knockout (*mc2r^KO^*) and wild type (WT) zebrafish. The table shows enriched GO molecular function terms, including their ID, description, gene ratio, background ratio, *P*-value, adjusted *P*-value, *q*-value, associated genes, and gene count.

**Supplementary Table 3.** Expression of pigment cell marker genes among differentially expressed genes between mc2r knockout (*mc2r^KO^*) and wild type (WT) zebrafish. The table includes Ensembl gene IDs, gene names, log fold change (logFC), P-value, and false discovery rate (FDR).
